# Motor Activity in Aging: An Integrated Approach for Better Quality of Life

**DOI:** 10.1155/2014/257248

**Published:** 2014-11-24

**Authors:** Lorenza Pratali, Francesca Mastorci, Nicola Vitiello, Annamaria Sironi, Amalia Gastaldelli, Angelo Gemignani

**Affiliations:** ^1^Institute of Clinical Physiology, National Research Council, Via Moruzzi 1, 56125 Pisa, Italy; ^2^The BioRobotics Institute, Scuola Superiore Sant'Anna, Polo Sant'Anna Valdera, Viale Rinaldo Piaggio 34, Pontedera, 56025 Pisa, Italy; ^3^Department of Surgery, Medical, Molecular and Critical Area Pathology, University of Pisa, Via Paradisa 2, 56100 Pisa, Italy

## Abstract

Old age is normally associated with stereotypical structural and physiological changes in the brain that are caused by deterioration in elementary cognitive, sensory, and sensorimotor functions as well as increased susceptibility to stress. These changes are connected with gait impairment and falls, especially among patients with common neurological diseases. Even in the absence of history of falling or when there is no physical injury after a fall, many older people develop a fear of falling that leads to restricted mobility, reduced activity, depression, social isolation, worsened metabolic disease, and increasing risk of cardiovascular morbidity and mortality. Although links between cognitive decline and age-associated brain changes have been clarified, relationships between gait disorders and psychophysiological alterations in aging are less well understood. This review focuses on two crucial elements of aged individuals with gait disorders: characteristic comorbidities in the elderly and the psychophysiological effects of physical exercise in the elderly with gait disorder. We propose an integrated approach to studying elderly subjects with gait disorder before starting a program of motor rehabilitation with wearable robotic devices, in order to investigate the effectiveness and safety of the ambulatory training.

## 1. Introduction

Our aging population is one of the most critical challenges of current industrialized societies. This issue will need to be tackled in the next few years since it threatens the sustainability of social welfare. Forty years from now, nearly 35% of the European population will be over 60, and hence it is urgent to provide solutions enabling our aging society to remain active, creative, productive, and—above all—independent, as underlined in the “*Report of Health Aging in America, 2007; Report of United Nations, 2009*.”

Aging is a complex phenomenon involving multiple biological pathways, from the microscopic (molecular and cellular) to macroscopic levels (tissues and organs) [1]. However, the epidemiological translation of these biological processes is quite univocal: accelerated aging diminishes healthy life expectancy. Age itself is an independent morbidity and mortality risk factor for various diseases, injuries, hospitalization, length of hospitalization, and adverse drug reactions [2, 3]. Gait disorders and lower-limb impairment are common and often cause devastating quality-of-life issues for aged individuals [4, 5]: several population-based studies have shown a 35% prevalence of gait disorders among people over 70 years old [6] and even over 85 years old. Immobility or hypomobility due to gait disorder in aging causes decreased survival, which can be attributed to a combination of fatal falls, reduced cardiovascular fitness, and death from an underlying disease [4, 6, 7]. Usually, even in the absence of a history of falling, nearly one-third of older adults experience a fear of falling that consequently leads to self-imposed restrictions in mobility, reduced activity, depression, social isolation, and loneliness [8]. In particular, falls lead to major fractures or head trauma, and it is expected that this could occur in 500,000 individuals by the year 2040, with an expected total annual cost in 2040 of 16 billion dollars [9]. Spontaneous walking speed normally decreases by about 1% per year from the age of 60 onward [10], and the observed decline of maximum walking speed is even greater. Many older people accept their gait difficulties as normal for their age; their doctors often support them in this view.

**Figure 1:**
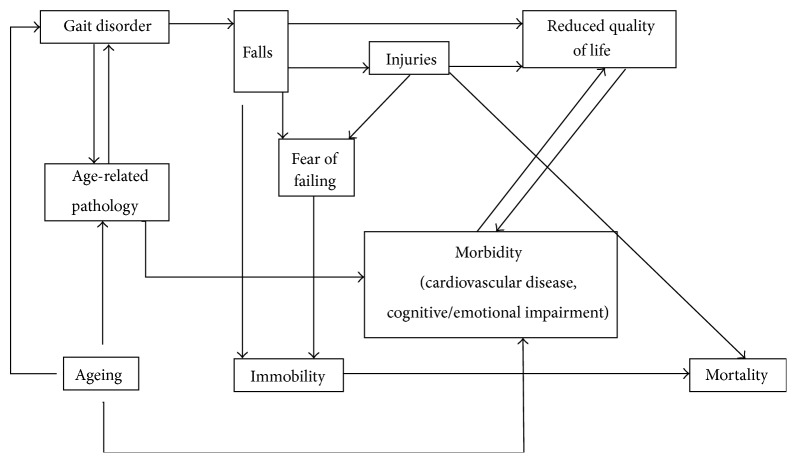
Association between aging and gait disorders. Adverse consequences of age-related gait disorders lead to reduced quality of life (depression, social isolation, and cognitive decline) and, in some cases, to mortality.

*Are Gait Disorders Truly an Inevitable Consequence of Aging Itself?* Recent findings have challenged this concept: 20% of very old individuals walk normally; hence, gait disorders are certainly not an inevitable feature of old age [11]. Senile gait disorders could thus be an early manifestation of underlying pathology, most notably subtle white/grey-matter changes, vestibular, visual, or oculomotor dysfunctions. Such disorders might alter gait directly but may also act in an indirect way by causing a subjective sensation of instability and insecurity, forcing individuals to adopt a more cautious gait [12–18]. The association between aging and gait disorders is thus a complex, yet, open issue, which is well summarized by the diagram in Figure 1, adapted from Snijders et al. [4]. Aging also affects the level of cognitive involvement in gait-related motion tasks. Walking is traditionally seen as an automatic motor task that requires little, if any, higher mental function. However, the safety and efficacy of normal walking only on rely not sensorimotor systems, but also critically depend on the interaction between the decisions of action with the cognitive dimension. In this regard, attention is a necessary cognitive resource for maintaining normal walking and there is evidence that cognitive and attention deficits are independently associated with postural instability and future falls [19]. A common situation where such integration is challenged is when people must walk while performing a secondary task. The ability to maintain normal walking while performing a secondary task has become the classic way to assess the interaction between cognition and gait [20]. In elderly people, this ability deteriorates because central resources decline. This leads to the fact that more mental effort is required in elderly people to perform locomotion. This further increases the complexity of the task or, dually, increases the chance of gait dysfunctions. In patients with overt disease, such as stroke or Parkinson's disease, gait deteriorates even more during dual tasking [21, 22].

This review focuses on two crucial elements of aged individuals with gait disorders: characteristic comorbidities in the elderly and the effects of physical exercise in elderly persons with gait disorder. We will emphasize the presence of different comorbidities in the elderly and the importance of a multidisciplinary approach to performing studies evaluating the beneficial effect of the regular exercise in this subset of subjects. This point represents the philosophy of our perspective program of motor rehabilitation where medicine (cardiology, metabolism, and neuropsychology) meets biomedical engineering (wearable robotic devices) with the unique aim of improving the quality of life of individuals with gait disorders, in order to reduce any related medical comorbidities.

## 2. Gait Disorders in the Elderly and Medical Comorbidities

The elderly present different comorbidities in association with gait disorders, causing a stressful condition. “Stress” is the consequence of an organism becoming incapable of effectively coping with internal or environmental stimuli. Higher vulnerability and lower resilience of the stressed organism to any kind of input indicate the stress condition as the first subclinical step towards the clear manifestation of a disease, characterized by a decreased ability to maintain homeostasis [23]. In particular, chronic stress may produce cognitive dysfunction in the elderly and may increase the rate of cognitive decline in Alzheimer's patients. Stressful lifestyles have been suggested to increase glucocorticoid levels in the brain and this is neurotoxic, affecting neuronal energy balance, producing a decline in cognitive function, and increasing susceptibility to insomnia, depression, cardiovascular disease, and diabetes [24].

### 2.1. Aging and Sarcopenia

Several reports have indicated an age-related loss of skeletal muscle, that is, sarcopenia [25–28]. Elderly subjects, even when not obese (i.e., with BMI < 30 kg/m^2^), have an increased percent fat mass compared to younger counterparts. The causes include medical, behavioral, and environmental factors that characterize aged individuals. The decreased muscle mass that occurs with aging has been extensively described in humans [29, 30]. It has been suggested that loss of muscle mass might be due to decreased fractional rate of muscle protein synthesis [31, 32]. However, this is not a consistently observed response. Volpi et al. [33, 34] have shown that under basal conditions the fractional rate of muscle protein synthesis was not impaired in apparently healthy elderly persons. Increased fasting plasma concentrations of essential amino acids (i.e., not synthesized by the human body) and in particular branched chain amino acids are often used as an indicator of protein catabolism. In the postprandial state, the availability of blood amino acids is a potent stimulus for muscle protein synthesis [35, 36] and essential amino acids are primarily responsible for this stimulatory effect [37]. In the elderly, the mechanisms associated with the stimulation of muscle protein synthesis by an elevation in blood essential amino acids are less responsive compared to younger subjects [38]. It is important to underline that sarcopenia is a reduction not only in muscle mass, but also in muscle function and can promote bone fragility due to bone mass loss and decreased bone strength. Muscle weakness, fear of falls, falls, and subsequent fractures are associated with concurrent sarcopenia and osteoporosis and lead to restricted mobility, loss of autonomy, and reduced life expectancy. The skeletal and muscular organ systems are tightly intertwined: the strongest mechanical forces applied to bones are indeed those created by muscle contractions that condition bone density, strength, and microarchitecture. For these reasons, a decrease in muscle strength is often associated with lower bone strength.

### 2.2. Aging and Energy Expenditure

Generally, older people tend to be increasingly sedentary; muscle disuse reduces energy requirements and appetite and the total energy expenditure (TEE) is therefore reduced. TEE is the amount of energy needed by anyone to meet their daily physical demands and has three components: (1) the amount of energy needed to maintain the body's needs at rest—basal energy expenditure—expressed as the basal metabolic rate (BMR); (2) diet-induced thermogenesis (DIT); (3) needs generated by the daily activity levels, which include physical activity grade [39]. The BMR represents the total body daily energy requirement (60% of TEE), which permits the function of all of the essential body systems: mainly brain, respiratory and cardiovascular function, and the work of the thermoregulatory system. Many factors can influence BMR, such as age, gender, body temperature, body weight, body composition, genetic factors, hormonal status, and pathological conditions. However, the main determinants of the BMR are fat-free mass (FFM), age, and gender [40, 41], which together account for about 80% of interindividual variability. The BMR is also associated with a decreased FFM and a lowered cellular metabolism. The FFM is the major determinant of BMR and comprises the various body organs and the skeletal muscle mass. The individual muscle mass in turn depends on age, nutrition (diet and nutritional state), and physical activity. Gender determines a lower BMR in females with respect to males with the same weight, height, and age. This difference begins at around the age of 3 and increases until puberty, when muscle mass increases in males whereas in females adipose tissue increases. BMR decreases with age, especially in the elderly, with decreased muscle mass and cellular metabolism. The DIT can be defined as the increase in energy expenditure above basal fasting level divided by the energy content of the food ingested and is commonly expressed as a percentage [42]. It accounts for 10% of TEE. DIT depends on the quality and the quantity of food introduced per meal: carbohydrates and fat have a lower thermogenesis (5–10%) with respect to protein (20–35%). DIT is related to the stimulation of energy-requiring processes during the postprandial period. Stimulation of adenosine triphosphate hydrolysis during intestinal absorption, initial metabolic steps, and nutrient storage are responsible for this food thermic effect [43]. Physical activity is the third component of the TEE: it depends on the individual's daily activity and constitutes 15–30% of TEE. Despite the demonstrated benefits of physical activity [44], it is well known that the vast majority of older adults are physically inactive and that the prevalence of inactivity increases with advancing age. Taking into account all the TEE components, aging is associated with a TEE decline disproportionately greater than the decline in daily energy intake. Collectively, these events can create a “positive” energy balance, with secondary gains in central and total body fat, and a subsequently higher risk of morbidity and mortality [44], also accompanied by reduced muscle mass. With the growth in the number and life expectancy of older adults, the numerous risks (e.g., disability, chronic disease, reduced functional abilities, and increased falls) [45, 46], associated with the prevalence of inactivity in old age, are potentially an enormous burden not only to the older adult himself, but also to society as a whole.

### 2.3. Aging and Cardiovascular Disease

Aging is typically associated with cardiovascular disease, with increased evidence of systemic atherosclerosis and also a decrease in cardiovascular performance [47, 48]. Atherosclerosis and its subsequent cardiovascular complications (myocardial infarction, stroke, and ischemic heart failure) are a major cause of death in the Western world. The risk factors of atherosclerosis are well known, including hypertension, diabetes, serum total and low-density lipoprotein cholesterol, and smoking. Increasing evidence indicates that aging is also an important risk factor for atherosclerosis and persists as an independent contributor when all other known factors are controlled. Premature or accelerated vascular aging can be promoted by cardiovascular risk factors, and cellular senescence is also observed in patients with atherosclerosis [49, 50]. Atherosclerosis is therefore a disease of both organic aging and cellular senescence par excellence. Cellular senescence impairs cell proliferation, resulting in irreversible growth arrest and impaired survival, due to an accumulation of nuclear and mitochondrial DNA damage, increased reactive oxidative species, and a proinflammatory state. Both vascular aging and cellular senescence are associated with increased expression of proinflammatory cytokines and adhesion molecules further promoting inflammation and also affecting the synthesis and maintenance of extracellular matrix proteins. Aging can be identified both by structural changes and by a number of senescence-associated biomarkers. However, major gaps in our knowledge exist as to whether small changes in these biomarkers reflect an important loss of function and how aged cells promote disease. Aging is also associated with a decline in cardiovascular performance, more apparent during physical stress than at rest. The hallmarks of cardiovascular aging are reduced maximal heart rate, ejection fraction, and, in most studies, reduced maximal cardiac output with stress test [48, 51]. The cardiovascular alterations that occur with aging in some ways parallel the changes that occur with deconditioning, including a decrease in maximal oxygen intake and maximal cardiac output. Several of the changes noted with aging are related to disuse and normalize with increased activity [52]. Although the effects of training on cardiovascular function are relatively well described in younger subjects, little is known regarding the changes that occur with training in older subjects and it has become clear that older subjects can adapt to exercise training [51, 53]. In elderly people, prevention of accelerated cell aging becomes a major therapeutic opportunity, so understanding the mechanisms contributing to changes typically associated with aging is crucial for both the prevention and development of treatment for age-related diseases.

### 2.4. Aging and Cognition, Emotion, and Sleep

Aging in humans is accompanied by stereotypical structural and physiological changes in the brain that sustain variable degrees of cognitive decline and sensorimotor dysfunction [54]. The modern neuroscientific point of view suggests that normal and especially pathological aging are not associated with cerebral regional atrophy or damage but rather with an alteration of large-scale integration between different neuronal pools, which may sustain a kaleidoscopic clinical picture, involving different aspects of brain function, from cognition to emotions, from motor to sensory, and from sleep to autonomic control [55]. Aged individuals who show delocalized activity show better cognitive performance than aged individuals with more localized activity, supporting the idea that delocalization may be a compensatory response or the effect of a residual neuronal or network plasticity [56]. These highly reliable changes occur gradually throughout middle age and are influenced by sex, age, social status, and educational background [57, 58]. The presence of residual plasticity may constitute a barrier against age-related neurodegenerative changes [59]. In general, functional imaging studies of the human brain have revealed that cognitive functions were less coordinated, suggesting a global loss of integration, responsible for the normal activities of daily living, such as walking, dressing, or driving a vehicle [60]. This lack of coordination, typical of the elderly, will result in a higher risk of falling [61] and decreased functional and social independence [62]. In contrast with the age-related cognitive changes, emotional behavior seems to be odd, varying from an enhanced to abnormal emotional reaction such as depression [63, 64]. Some clinical findings show that subjects experience less negative effect as they get older and can restore their good mood after a negative mood more rapidly than younger adults [65]. This peculiar emotional behavior seems to support the hypothesis that elderly have better control of their emotions, despite their cognitive decline. One possible explanation for this incongruence was provided by Cacioppo et al. [66], who showed that age-related decline in the functioning of the amygdala leads to positive effects on mood and, more generally, on emotional behavior. This amygdala-related decline selectively diminishes emotional arousal in response to negative stimuli but not to positive ones.

On the contrary, a large body of the literature indicates that aging is a vulnerable condition for developing clinical depression [67]. Furthermore, this link seems to be strongly sustained by age-related sleep changes or alterations such as insomnia [68]. Not only depression but also a large number of physical health conditions such as pain syndromes (fibromyalgia, osteoarthritis), primary pulmonary problems (chronic obstructive disease, bronchitis, and asthma), neurological disturbances (cerebrovascular accidents), and neurodegenerative disorders such as Alzheimer's disease and Parkinson's disease show a mutual relationship with sleep problems [69]. In this regard, changes in sleep architecture over the lifespan are well known [70]. From a homeostatic point of view, decreased slow-wave sleep (SWS) is a crucial factor in aging. It has been shown that the decrease in amplitude of SWS is a result of brain atrophy over time. Also, sleep spindles and K-complexes become less numerous, and the frequency of the spindles becomes slower as we age [71]. All these changes, according to Tononi and Cirelli's synaptic homeostatic hypothesis of sleep function [72], yield to a dramatic disruption of sleep-related plastic phenomena, boosting cognitive decline. It is more difficult to identify a link with depression. Adult-age depression is always characterized by decreased SWS (mainly in the first sleep cycle) [73], typically associated with an increase in hypothalamic-pituitary-adrenal (HPA) axis activity or changes in neuronal plasticity (mainly in frontal areas) [74]. These two abnormal conditions are the stigmata of aging. In addition, aging is often associated with social isolation, and this condition represents a trigger factor for depression and related pathoplastic changes (decreased SWS, abnormal activation of HPA axis, and impoverishment of synaptic plasticity) [8]. Social isolation is often associated with hypomobility. It is well known that aged individuals reduce their motor activity, especially when a gait disorder is present. Motor activity is a boosting mechanism for brain plasticity, hippocampal neurogenesis, and SWS [75]. Thus, alterations in motor activity in older people can enhance their vulnerability to cognitive decline and depression. Along these lines, it has been shown that motor programs in older individuals with depression and insomnia improve the overall clinical picture. We also have to take into consideration that drugs used to manage chronic illnesses may also contribute to developing insomnia and depression in elderly people [76].

## 3. Physical Inactivity and Aging: A Dangerous Pair

From a physiological point of view, physical inactivity can be considered an aggression and induces the same mechanisms of stress response. This inappropriate stress response and the induced changes become risk factors for chronic disease. Immobilization is associated with decreased muscle glucose utilization (by increasing muscle insulin resistance) and triggers muscle atrophy. Both of these effects decrease further energy consumption by unused muscles. Energy is reallocated to the liver, which increases lipid production. Lipids are preferentially stored in central fat tissue. Central adipocytes are metabolically active when loaded with fat and they both produce inflammatory molecules and decrease secretion of anti-inflammatory adiponectin [77]. Moreover, the number of macrophage cells rises in fat tissue along with fat deposits. These macrophages become activated and produce proinflammatory cytokines [78, 79]. Stress response, high glucose levels, and proinflammatory cytokines increase blood coagulation and platelet aggregation and inflammation. These induced mechanisms are also critical for determining cardiovascular disease; the traditional view of atherosclerosis as a lipid storage disease crumbles in the face of extensive and growing evidence that inflammation participates centrally in all stages of this disease, from the initial lesion to the end-stage thrombotic complications [77]. Recent findings confirm that physical activity induces an increase in the systemic levels of a number of cytokines and chemokines with anti-inflammatory properties. It is possible that regular physical exercise exerts anti-inflammatory activity, since the interaction between contracting muscle and the other tissues and the circulating cells is mediated through signals transmitted by “myokines” produced with muscle contractions. To date, the list of myokines includes IL-6, IL-8, and IL-15. During muscle contractions IL-1 receptor antagonist and sTNF-R, molecules that contribute to anti-inflammatory actions, are also released. In spite of some discrepancies, analysis of available research seems to confirm the efficacy of regular physical training as a nonpharmacological therapy targeting chronic low-grade inflammation. Given this, physical exercise could be considered a useful weapon against local vascular and systemic inflammation in atherosclerosis. Several mechanisms explain the positive effect of chronic exercise; nevertheless, these mechanisms do not fully explain all pathways by which exercise can decrease inflammation and endothelial dysfunction and hence modulate the progression of the underlying disease progress [80]. Therefore, physical activity and aging have opposite effects on a clear end point: healthy life expectancy. Several epidemiological studies have established the benefits of physical activity for improving both life expectancy with or without disease and life expectancy in good health (healthy life expectancy) of elderly individuals. Beneficial associations between physical activity and mortality have been found in populations with various age distributions and in various geographical areas. A recent meta-analysis reviewed relevant studies of the dose-response relationship of nonvigorous physical activity and all-cause mortality [81]. These authors found that 2.5 h/week (equivalent to 30 min daily of moderate intensity activity 5 days a week) compared with no activity was associated with a reduction in mortality risk of 19% (95% confidence interval (CI) 15–24), while 7 h/week of moderate activity compared with no activity reduced mortality risk by 24% (95% CI 19–29). The largest benefit was found when moving from no activity to low levels of activity, but even at high levels of activity benefits accrue from additional activity. Moreover, recently a case-control study on the effect of the level of physical activity and the risk of myocardial infarction was published [82]. The INTERHEART study enrolled 29,000 people from 52 countries in Asia, Europe, The Middle East, Africa, Australia, and North and South America and examined how physical activity both at work and during leisure time is related with cardiovascular risk. It shows that mild to moderate physical activity at work and any level of physical activity during leisure time reduce the risk of heart attack, independent of other traditional risk factors, in men and women of all ages, in most regions of the world and in countries with low-, middle-, or high-income levels. Inactivity is a universal cardiovascular and metabolic risk factor and physical activity remains the single most neglected therapeutic intervention worldwide. The protective role of physical activity in relation to cardiovascular and metabolic disease is accompanied by an important and well-documented involvement of motor performance in cognitive function. In particular, it is interesting to note that, among older adults, cognitive and movement disorders such as coordination difficulty [83], increased variability of movement [84], and slowing of movement [85] cooccur more frequently than what would be expected by chance, and the occurrence of either disorder raises the risk of onset of the other. Research investigating the effects of exercise on older adults has primarily focused on brain structural and functional changes with relation to cognitive improvement. In particular, several cross-sectional and intervention studies have shown a positive association between physical activity and cognition in older persons [86] and an inverse correlation with cognitive decline and dementia [87]. Older adults enrolled in a 6-month aerobic fitness intervention increased brain volume in both gray matter (anterior cingulate cortex, supplementary motor area, posterior middle frontal gyrus, and left superior temporal lobe) and white matter (anterior third of corpus callosum) [88]. In addition, Colcombe and colleagues showed that older adults with higher cardiovascular fitness levels are better at activating attentional resources, including decreased activation of the anterior cingulated cortex. One of the possible mechanisms by which physical activity may benefit cognition is that physical activity maintains brain plasticity, increases brain volume, stimulates neurogenesis and synaptogenesis, and increases neurotrophic factors in different areas of the brain, possibly providing reserve against later cognitive decline and dementia [89, 90].

## 4. Methodologies and Techniques for Investigating Cardiovascular, Metabolic, and Cognitive Response Elicited by Motor Rehabilitation

**Figure 2:**
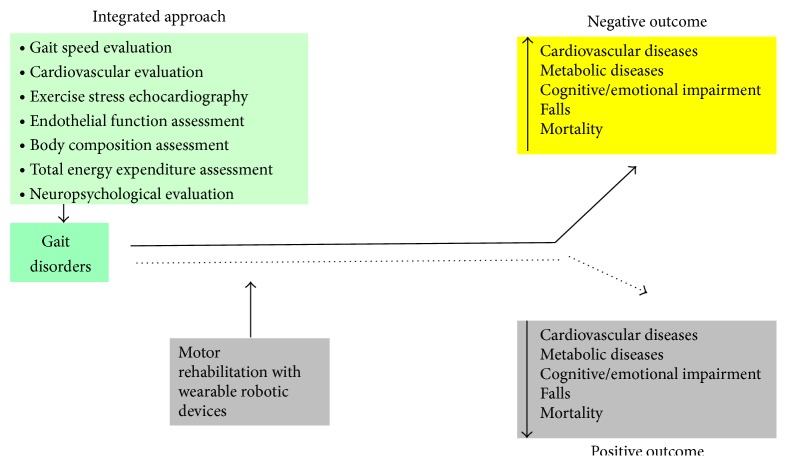
Integrated approach (cardiovascular, metabolic, and cognitive) to studying the possible outcome of motor rehabilitation.

One of the main characteristics of the elderly population is its heterogeneity, and older people in the same age range show a wide variation in their risk of disability, cardiac and metabolic disease, cognitive impairment, insomnia, depression, hospitalizations, institutionalization, falls, and mortality. To prevent these adverse outcomes, population-based intervention programs should target the population at risk. Therefore, a feasible and valid screening tool available for research and clinical settings is required to identify target populations. Here we propose an integrated approach to study older subjects with gait disorder before starting a program of motor rehabilitation with wearable robotic devices to investigate the effectiveness and safety of the ambulatory training (Figure 2).

## 5. Conclusion and Future Directions

The needs of elderly people with lower-limb impairment motivated us to start a collaborative research project aimed at developing an assistive robot for the functional support of lower-limb motion. This project intends to research means of cognitive control in a multidegree-of-freedom system and to develop know-how on how humans can interface with a semiautonomous robotic device that supports a disabled person in executing locomotion-related tasks (e.g., walking, stair climbing), including transients (e.g., start, stop, sit-to-stand, etc.), in a real-life unstructured environment. A multidisciplinary team (engineer, medical doctor, and therapists) will collaborate on this project to validate the new assistive robot and to investigate the acute and chronic effect of exercise in older persons with gait disorders, assisted by a wearable robot for the lower limbs.
